# Complete Coding Sequence of Lumpy Skin Disease Virus Isolated from Kinmen Island, Taiwan, in 2020

**DOI:** 10.1128/mra.01204-21

**Published:** 2022-03-17

**Authors:** Chih-Wei Huang, Lu-Jen Ting, Yu-Pin Liu, Yu-Ju Lin, Fan Lee, Chwei-Jang Chiou

**Affiliations:** a Animal Health Research Institute, New Taipei City, Taiwan; Queens College, CUNY

## Abstract

We reported the complete coding sequence of a lumpy skin disease virus (LSDV) isolated from cattle from Kinmen, Taiwan, in 2020. The nucleotide sequence of LSDV/KM/Taiwan/2020 was most closely related to strains from an outbreak in China and Vietnam in 2020 and clustered within the vaccine or vaccine-derived clade.

## ANNOUNCEMENT

Lumpy skin disease virus (LSDV) is an emerging pathogen of the family *Poxviridae*, having spread over the past 10 years from Africa and the Middle East into southeastern Europe, the Caucasus, Russia, and, more recently, Asia (1–8). It will cause an economic impact on the cattle industry in the invaded regions (9–10). Here, we report the complete coding sequence of an LSDV isolate obtained from the first outbreak in Kinmen Island of Taiwan in 2020.

The LSDV/KM/Taiwan/2020 isolate, recovered from skin lesions of affected cattle, was grown in primary sheep testis cells following the protocol of the World Organization for Animal Health (OIE) (11). DNA was purified from cell culture supernatant harvested when cytopathic effects were observed, using a MagNA Pure compact nucleic acid isolation kit I (Roche Diagnostics, Mannheim, Germany). A paired-end sequencing library was constructed with a Nextera DNA Flex library prep kit (Illumina, San Diego, CA, USA) following the manufacturer’s protocols. Sequencing was performed using a 500-cycle (2 × 250-bp paired-end) MiSeq reagent kit version 2 (Illumina, San Diego, CA, USA) with an MiSeq sequencer. Default parameters were applied for all programs unless specified. Bases lower than Q30 were trimmed using BBDuk implemented in Geneious Prime version 2020 (https://www.geneious.com). *De novo* assembly was performed using Geneious assembler with low-sensitivity setting and 3% maximum mismatches per read. The longest consensus sequence was identified as LSDV using BLASTN search. The assembled sequence was further checked via mapping. Open reading frames (ORFs) were predicted with initial codon ATG by ORF Finder. Complete genomes of selected wild and vaccine LSDV strains were aligned using MAFFT version 7 (12, 13). Maximum-likelihood phylogeny was reconstructed using IQTREE version 1.6.12 (14, 15) with 1,000 replicates of ultrafast bootstrap approximation (16) for branch support assessment. Phylogeny was visualized by FigTree version 1.4.4 (http://tree.bio.ed.ac.uk/software/figtree/).

**FIG 1:**
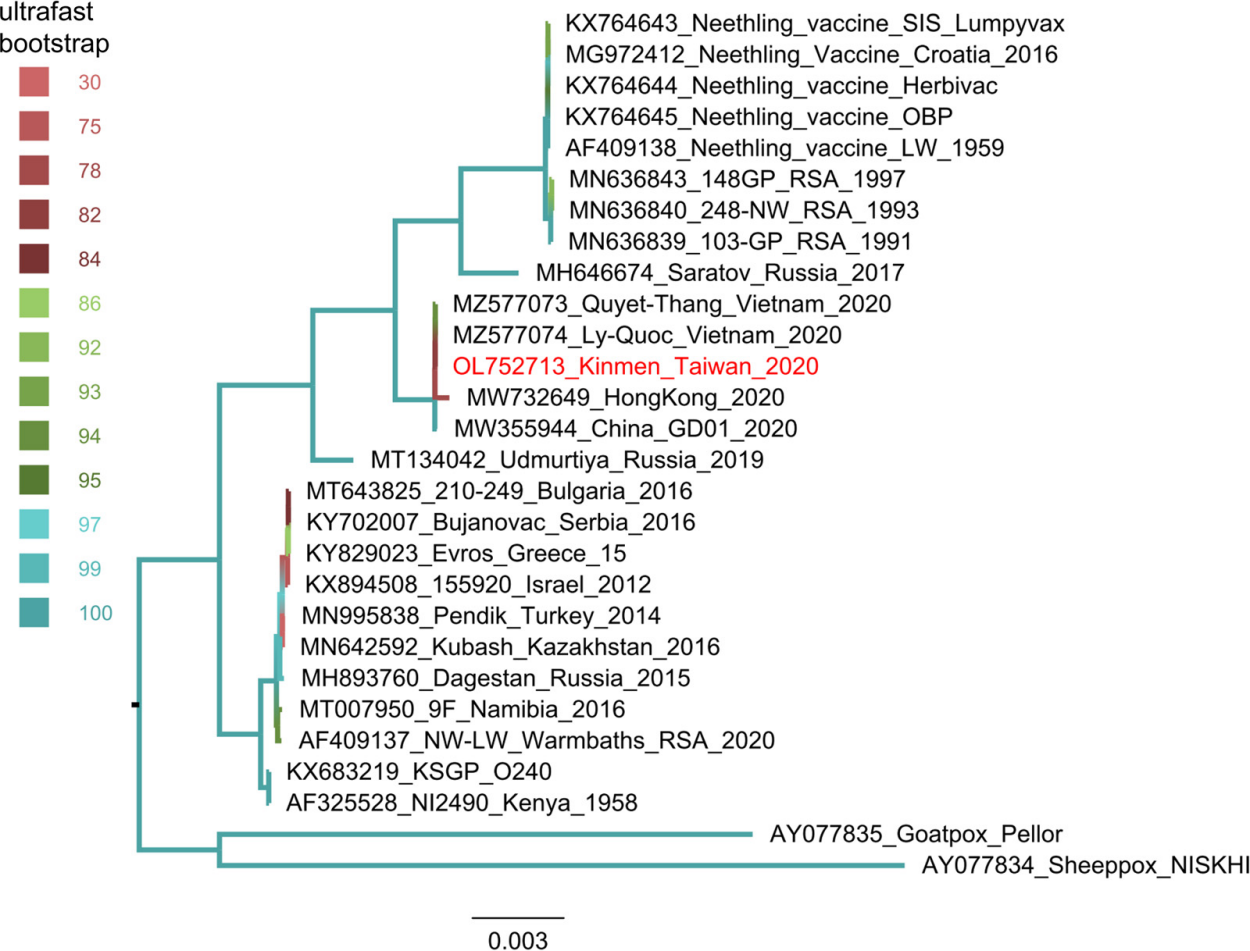
Maximum-likelihood phylogeny of lumpy skin disease virus based on complete genomic sequences. The color of branch indicates the branch support based on 1,000 replicates of ultrafast bootstrap approximation.

In total, 2,552,662 reads were acquired (SRA accession number SRX14182446). The assembled genome of LSDV/KM/Taiwan/2020 was 150,822 bp with 25.9% GC content (GenBank accession number OL752713). The average sequencing depth was 380.8×. The genome was 99.99% identical to four Vietnam isolates (GenBank accession numbers MZ577073 to MZ577076). Two indels and two single nucleotide polymorphisms (SNPs) were identified among the genome alignment of LSDV/KM/Taiwan/2020 and two Vietnam isolates (MZ577073 and MZ577074) (Table 1). One point mutation caused an amino acid change from leucine to serine that encoded the viral membrane protein of the entry-fusion complex component (Table 1). Maximum-likelihood phylogeny showed that the LSDV/KM/Taiwan/2020 isolate clustered with strains that were associated with outbreaks in China and Vietnam in 2020 and close to vaccine or vaccine-derived strains (Fig. 1).

**TABLE 1:** Mutations in viral genome of the LSDV/KM/Taiwan/2020, which is the first lumpy skin disease virus isolated on Kinmen Island, Taiwan, in 2020

Region in the alignment (bp)^*a*^	Mutation type	Mutation	Note
275–289	15-bp deletion	TAAGTGGAAGCCAAT	
150650–150721	72-bp insertion	TTATTAGGTTTAATTGGCTTCTACTTAATTGGCTTCCACTTATTAGGTTTAATTGGCTTTTTATAATTAGGT	
64585	Mutation	T→C	Leu→Ser in entry-fusion complex component (viral membrane protein)
150734	Mutation	C→T	

a Genome alignment of LSDV/KM/Taiwan/2020 (GenBank accession number OL752713), 20L42_Quyet-Thang/VNM/20 (GenBank accession number MZ577073), and 20L43_Ly-Quoc/VNM/20 (GenBank accession number MZ577074).

This work was performed at the Animal Health Research Institute, which is the national veterinary laboratory in Taiwan, and no ethical approval was required for the work carried out.

### Data availability.

Raw reads were deposited in SRA under accession number SRX14182446. The assembled genomic sequence of the isolate LSDV/KM/Taiwan/2020 has been deposited in GenBank under accession number OL752713.
